# Quadratic Characteristics of Environment Induced Voltage Shot Noise in Josephson Junctions

**DOI:** 10.1038/s41598-017-03790-x

**Published:** 2017-06-15

**Authors:** Jyh-Yang Wang, Tao-Hsiang Chung, Teik-Hui Lee, Chii-Dong Chen

**Affiliations:** Institute of Physics, Academia Sinica, Taipei, 115 Taiwan

## Abstract

We investigate theoretically and experimentally the environment-induced voltage shot noise in current biased Josephson junctions induced by phase particle tunneling. Quantum mechanical treatment based on the Caldeira-Leggett model with tight-binding formulation in local Wannier bases gives a clear picture of the voltage shot noise. A universal form of the zero-frequency noise spectrum is obtained, which exhibits a quadratic dependence on the mean voltage in small bias region. The quadratic dependence is verified experimentally on junctions covering a wide range of parameters, and is found also in junction arrays of various array sizes.

## Introduction

The shot noise is originated from the granularity of the particles^1–3^. However, in tunneling devices with negligible level spacing, the noise in tunneling current induced by particle-environment interaction is classified as thermal rather than shot noise. We argue that for systems with the level spacing much greater than the thermal energy, the particle interacting with environment can induce a new class of shot noise. In the case of negligibly small level spacing, the shot noise occurs due to random scattering of incoming electrons by the tunnel barrier, and the thermal noise is attributed to the interaction with the environment or thermal bath because of the thermal distribution of the energy spectrum in the two leads. In the case of discrete spectrum, the environment-induced stochastic process would play an important role and produce shot noise. This picture of particle-environment interaction induced stochastic process is valid for all kinds of particles that carry electric charges or magnetic fluxes. Here, we consider phase particle tunneling induced shot noise in Josephson junctions (JJs). Specifically, these phase particles tunnel between neighboring washboard potential valleys in which the separation of energy levels is defined by the Josephson plasma frequency, and the shot noise would reveal in the measured voltage noise. This environment-particle interaction is a new source of shot noise. We further show that this voltage shot noise will have a quadratic dependence on the mean voltage in the small bias region. This is different from the familiar linear dependence of the shot noise in electronic tunnel devices.

Although there have been several studies on the voltage noise of current-biased JJs in the phase regime^4–9^, early theoretical and experimental works mainly focused on the case in which the phase particle is driven in the running state or the phase diffusion state^4–6^, where the shot noise picture based on quantized phase change is invalid. The voltage noise is analyzed by using the Langevin equation and is interpreted as the response induced by the fluctuating current of resistive components of the circuit. A linear trend of the voltage noise with the mean voltage is found in the phase diffusion regime and a decreasing trend in the running state regime. A recent experimental study on phase-slip from a metastable state to a resistive state in nano-scaled weak links showed universality of higher moments in the tunneling counting statistics^10^. Using full counting statistics derived from the Langevin equation, more recent theoretical investigations^7–9^ explored the region where the phase particle tunneling (phase slip) picture emerges. However, the considered thermal energy is greater than the plasma energy. Consequently, the shot noise is smeared by the thermal distribution of the available tunneling levels. In order to investigate the environment-induced shot noise, we study both theoretically and experimentally the voltage noise in current-biased Josephson junctions at temperatures well below the plasma energy. The granularity required for the shot noise is granted by the fact that the voltage across JJs changes by a fixed amount for each tunneling event. However, this alone does not warrant the existence of voltage shot noise, because it also requests level spacings greater than the thermal energy to prevent tunneling stochasticity induced by thermal occupation distribution of the energy levels. Study on this issue relies on full quantum mechanical treatment, in which both voltage *V* and phase difference φ are treated as operators rather than classical variables. We show that the environment induced stochastic tunneling does not cause intra-well thermal agitation, and is responsible for the voltage shot noise. A dual case of the present current biased JJ circuit is a voltage biased JJ in an inductive environment, with which the Bloch band dynamics such as charge localization were studied^11^.

## Theoretical analysis on the voltage noise

**Figure 1:**
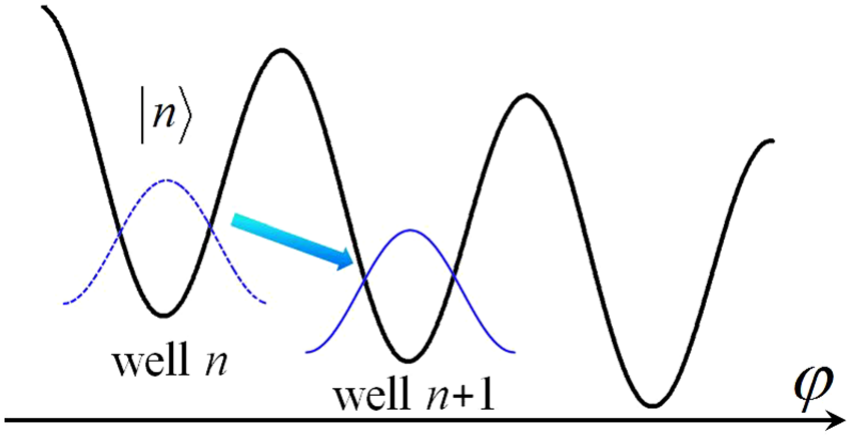
Schematic diagram of phase particle tunneling. Quantum mechanical description of a phase particle tunneling between neighboring valleys in the tilted washboard potential. The tunneling is the transition from a Wannier state $|n\rangle$ to the neighboring state $|n+1\rangle$.

The treatment starts from the relationship between the voltage noise and phase particle tunneling in a current biased JJ. We consider a JJ with the Josephson energy *E*_J_ much greater than the Cooper pair charging energy (*E*_J_ $\gg$ *E*_CP_) in the low temperature limit (*ħω*_p_ $\gg$ *k*_B_ *T*). Here, the JJ plasma frequency is $\hslash {\omega }_{{\rm{p}}}=\sqrt{2{E}_{{\rm{J}}}{E}_{{\rm{CP}}}}$. Also, we assume that the bias current *I*_b_ is much smaller than the critical current *I*_C_ to prevent the JJ from entering the running state. Under these conditions, the phase particle is highly localized at one of potential valleys and can only tunnel to the neighboring ones without the intra-well thermal agitation and inter-well thermal hopping. As illustrated schematically in Fig. 1, the phase particle localized in well *n* is presented by the Wannier state $|n\rangle$, which is approximately the ground state of each well. The tunneling process is understood as the transition of the phase particle from state $|n\rangle$ to $|n\pm 1\rangle$.

To find out the relationship between voltage noise and phase particle tunneling, explicit forms of the tunneling Hamiltonian and the voltage operator describing the voltage across the JJ are required. According to the model proposed by Di Macro *et al*.^12, 13^, the total Hamiltonian of an open quantum system consisting of a JJ coupled to the circuit environment is

1 $${\hat{H}}_{{\rm{tot}}}=-{{\rm{\Delta }}}_{0}\sum _{n}|n+1\rangle \langle n|+|n\rangle \langle n+1|-\frac{\hslash }{2e}{I}_{{\rm{b}}}\hat{\phi }+\frac{1}{2}\sum _{\alpha }[\frac{{\hat{Q}}_{\alpha }^{2}}{{C}_{\alpha }}+{(\frac{\hslash }{2e})}^{2}\frac{{({\hat{\phi }}_{\alpha }+\hat{\phi })}^{2}}{{L}_{\alpha }}].$$

The first term on the right hand side is the phase particle tunneling term, which describes the JJ Hamiltonian in the tight-binding approximation for the lowest band. The second band as compared to the lowest band has a contribution of the order of $\exp (-\hslash {\omega }_{{\rm{p}}}/{k}_{{\rm{B}}}T)$ and is negligible at low temperature limit, *k*_B_ *T* $\ll$ *ħω*_p_. The tunneling strength ${{\rm{\Delta }}}_{0}=4\sqrt{{E}_{{\rm{J}}}{E}_{{\rm{CP}}}/\pi }{(2{E}_{{\rm{J}}}/{E}_{{\rm{CP}}})}^{1/4}{{\rm{e}}}^{-4\sqrt{2{E}_{{\rm{J}}}/{E}_{{\rm{CP}}}}}$ is the bandwidth of the lowest Bloch band in the zero bias. The second term describes the current driving, and the last term describes the environment and its interaction with JJ. Based on the Caldeira-Leggett treatment^14^, the environment is modeled as a set of infinite number of quantum harmonic oscillators with corresponding conjugated pairs $({\hat{Q}}_{\alpha },{\hat{\phi }}_{\alpha })$, and the eigenfrequencies are characterized by inductances *L*_*α*_ and capacitances *C*_*α*_. The voltage operator $\hat{V}$, defined as $\frac{\hslash }{2e}\frac{{\rm{d}}\hat{\phi }}{{\rm{d}}t}$, is $\frac{1}{2e{\rm{i}}}[\hat{\phi },{\hat{H}}_{{\rm{tot}}}]$, which gives the following explicit form:

2 $$\hat{V}=-{\rm{i}}({{\rm{\Phi }}}_{0}{{\rm{\Delta }}}_{0}/\hslash )\sum _{n}|n+1\rangle \langle n|-|n\rangle \langle n+1|,$$

where Φ_0_ is the magnetic flux quantum. Utilizing the similarity between this form and the tunneling form for electric current operator, the mean voltage $\bar{V}$ and the zero frequency noise spectrum ${S}_{{\rm{V}}}(0)$ can be readily expressed in terms of tunneling rate ${{\rm{\Gamma }}}_{n\to n+1}$(${{\rm{\Gamma }}}_{n+1\to n}$) for phase particles^15^:

3 $$\{\begin{array}{c}\bar{V}{={\rm{\Phi }}}_{0}({{\rm{\Gamma }}}_{n\to n+1}-{{\rm{\Gamma }}}_{n+1\to n})\\ {S}_{{\rm{V}}}(0)=2{{{\rm{\Phi }}}_{0}}^{2}({{\rm{\Gamma }}}_{n\to n+1}+{{\rm{\Gamma }}}_{n+1\to n})\end{array}.$$

In these equations, we note that Φ_0_ indicates a phase change of 2*π* caused by each tunneling and reveals the granularity required for the voltage shot noise.

In order to study the influence of the environment on the tunneling rates, we adopted the *P*(*E*) theory in which the tunneling term is considered as a perturbation in the golden rule^16^. The solutions for forward and backward tunneling rates are given below:

4 $$\{\begin{array}{c}{{\rm{\Gamma }}}_{n\to n+1}({I}_{{\rm{b}}})=\frac{2\pi }{\hslash }{{{\rm{\Delta }}}_{0}}^{2}P({I}_{{\rm{b}}}{{\rm{\Phi }}}_{0})\\ {{\rm{\Gamma }}}_{n+1\to n}({I}_{{\rm{b}}})={{\rm{\Gamma }}}_{n\to n+1}({I}_{{\rm{b}}}){{\rm{e}}}^{-\beta {I}_{{\rm{b}}}{{\rm{\Phi }}}_{0}}\end{array},$$

where $\beta =1/({k}_{{\rm{B}}}T)$ and the function

5 $$P(E)=\frac{1}{2\pi \hslash }{\int }_{-\infty }^{\infty }{\rm{d}}t\cdot \exp [J(t)+{\rm{i}}Et/\hslash ]$$

is the probability density describing the rates of particle tunneling involving exchange of energy *E* with the environment. The environment charge-charge correlation function *J*(*t*) is ${(\pi /e)}^{2}{\sum }_{\alpha }\langle {\hat{Q}}_{\alpha }(t){\hat{Q}}_{\alpha }(0)-{\hat{Q}}_{\alpha }^{2}(0)\rangle$ ^13^. Given the impedance *Z*(*ω*) seen by the junction, *J*(*t*) can be further expressed as follows:

6 $$J(t)=2{R}_{{\rm{Q}}}{\int }_{-\infty }^{\infty }{\rm{d}}\omega \frac{\mathrm{Re}[1/Z(\omega )]}{\omega }\frac{{{\rm{e}}}^{-{\rm{i}}\omega t}-1}{1-{{\rm{e}}}^{-\beta \hslash \omega }}.$$

As can be seen in Eq. (4), the influence of the environment on the tunneling rate is given by $P({I}_{{\rm{b}}}{{\rm{\Phi }}}_{0})$, which according to Eq. (5) is just the spectrum of the exp[*J*(*t*)] at the frequency given by ${I}_{{\rm{b}}}{{\rm{\Phi }}}_{0}/2\pi \hslash$. Because there is no contribution from the thermal distribution of the intra-well energy to the tunneling rate even though the particle-environment interaction exists, the voltage noise is classified as shot noise. To better appreciate this point, let us examine the case of electron tunneling in a system with level spacing smaller than thermal energy such that the influence of the environment brings in thermal fluctuations of particle energy. In this situation, the charge tunneling rate of voltage-biased tunnel junctions given by *P*(*E*) is a double integral of $f(E)[1-f(E^{\prime} +e{V}_{{\rm{b}}})]P(E-E^{\prime} )$ over energy *E* and *E′*. Since this rate is governed by the thermal occupation of lead electron brought in by the Fermi function *f*(*E*), the shot noise is hidden by the thermal stochasticity.

The imbalance in the bidirectional tunneling process described by the Boltzmann factor ${{\rm{e}}}^{-\beta {I}_{{\rm{b}}}{{\rm{\Phi }}}_{0}}$ in Eq. (4) is originated from the detailed balance symmetry of *P*(*E*), namely, $P(-E)=P(E){{\rm{e}}}^{-\beta E}$. Due to this imbalance in the tunneling rate, the voltage shot noise in Eq. (3) has a universal form:

7 $${S}_{{\rm{V}}}(0)=2{{\rm{\Phi }}}_{0}\bar{V}\,\coth (\beta {I}_{{\rm{b}}}{{\rm{\Phi }}}_{0}/2).$$

**Figure 2:**
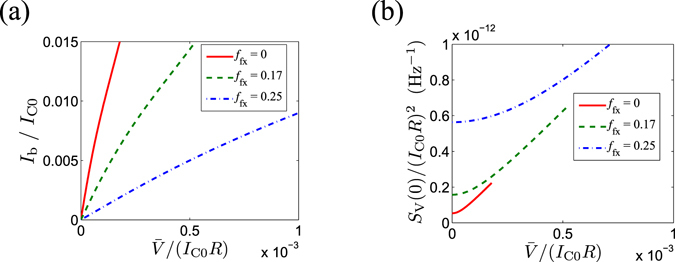
Numeric calculations based on the *P*(*E*) theory. (**a**) Mean voltage $\bar{V}$ as a function of bias current *I*_b_/*I*_C0_, with *I*_C0_ the zero-flux critical current of JJ. (**b**) Zero-frequency spectrum of voltage noise *S*_V_(0) as a function of mean voltage $\bar{V}$. The curves in both plots are shown for three filling factors: *f*_fx_ = 0, 0.17 and 0.25. The mean voltage $\bar{V}$ in (**a**) is calculated using equations (3–6) and the noise *S*_V_(0) is calculated using Eq. (7). In the calculation, *Z*(*ω*) = *R* + j*ωL* is used, where *R* = 60 Ω and *L* = 0.8 μH.

Accordingly, the mean voltage $\bar{V}$ is calculated as a function of bias current *I*_b_ (Fig. 2(a)), and the voltage noise *S*_V_ is calculated as a function of mean voltage $\bar{V}$ (Fig. 2(b)). Both plots show curves for three different flux filling factors *f*_fx_ defined as Φ_ext_/Φ_0_. Note that the Josephson energy *E*_J_ can be modulated periodically by Φ_ext_ from its zero-field value *E*_J0_ as *E*_J_(*f*_fx_) = *E*_J0_cos(π*f*_fx_). We note that $\bar{V}$ increases with *I*_b_ in an approximately linear manner in the voltage range of interest, namely, $\bar{V}\approx {R}_{0}{I}_{{\rm{b}}}$. In this range, the voltage noise in Eq. (7) can be approximated by using the Taylor expansion as

8 $${S}_{{\rm{V}}}(0)\approx \frac{4{R}_{0}}{\beta }+\frac{\beta {{\rm{\Phi }}}_{0}^{2}}{3{R}_{0}}{\bar{V}}^{2}.$$

Therefore, *S*_V_(0) displays a quadratic feature in small $\bar{V}$ region, as can be seen in Fig. 2(b), which is a general feature of the environment induced shot noise.

## Experiment setup and measurement results

**Figure 3:**
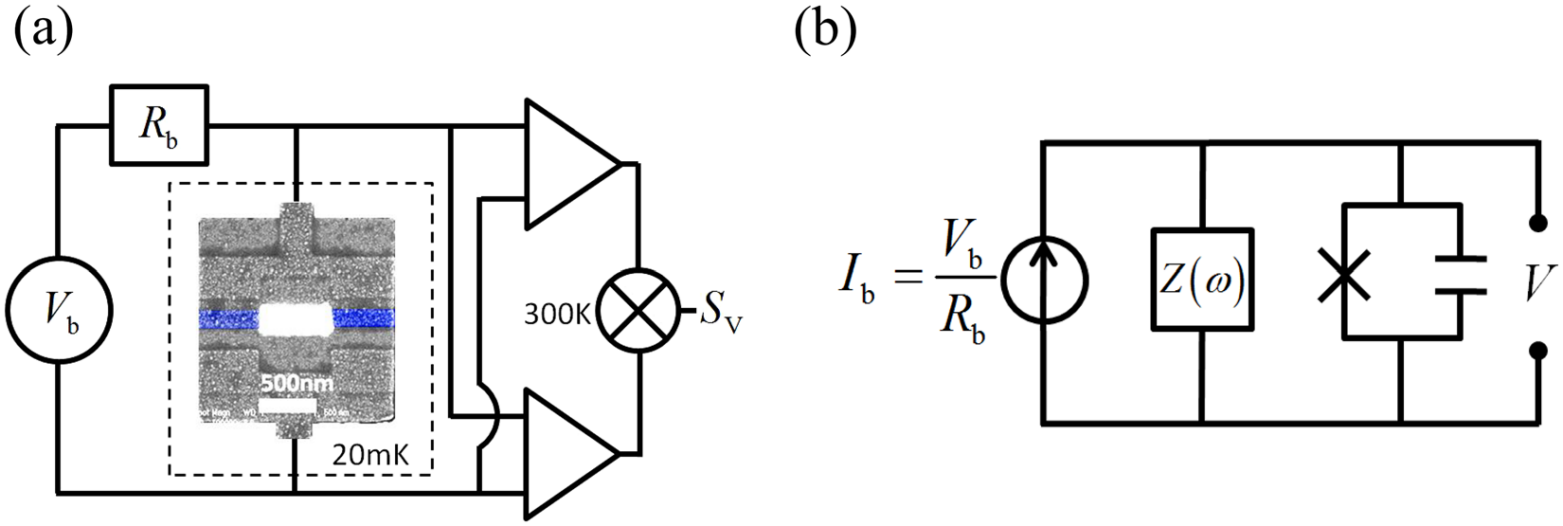
Experiment setup (**a**) Noise measurement circuit diagram including an SEM image of the JJ under study. The junction area is marked by the blue rectangles. The junction is current biased via a series resistor *R*_b_ of 1 MΩ, and *S*_V_(0) is measured with two voltage amplifiers whose outputs are cross-correlated. (**b**) The equivalent measurement circuit, in which *Z*(*ω*) represents a shunting impedance seen by the JJ.

Experiments were performed to support our analysis. The experiment setup is shown in Fig. 3(a), where the JJ was made in a SQUID form to have the *E*_J_ tunable by externally applied flux. The Al-AlO_x_-Al junctions on SiO_2_/Si substrate were fabricated by using e-beam lithography and double-angle deposition techniques. The current bias was realized by inserting a resistor between the voltage source and JJ with the resistance value (1 MΩ) much greater than that of the JJ. Several junction devices were investigated, and their junction parameters, including charging energy for Cooper pairs, *E*_CP_ , and zero-flux Josephson energy *E*_J0_ are listed in Table 1, and they all fulfill the condition that *E*_J0_>>*E*_CP_ . The measurement was performed in a dilution refrigerator at a base temperature *T* of about 20 mK. With such energy scale of *E*_CP_ , *E*_J0_, and *T*, the quantum tunneling of phase particles prevails the thermal agitation and/or hopping process. The DC measurement was carefully carried out in a four-probe scheme with symmetrical circuit configuration to take full advantages of common-mode noise rejection. The noise measurement was performed using the cross correlation techniques. Two ultra-low-noise amplifiers NF LI-75A with a fixed gain of 100 were used to measure the voltage noise across the devices in the frequency range between 1 kHz and 10 kHz, where the 1/*f* noise was negligibly small. The two amplifiers were operated at room temperature and were powered by independent sets of power supply to minimize any possible crosstalk. The voltage signals from the amplifiers were cross-correlated using a dynamic signal analyzer SR785. This noise measurement scheme was verified by well-established characteristics such as temperature-dependent Johnson-Nyquist noise power spectrum of resistors as well as the current shot noise Fano factor of JJs. Details of these test measurements are given in the Supplemental Material. The equivalent measurement circuit is shown in Fig. 3(b), in which the voltage source is transferred into a current source using the Norton’s theorem. The influence of the environment on the junction is depicted as a shunting impedance *Z*(*ω*) on the order of free space impedance at nonzero frequency^17, 18^, in which the contribution of *R*_b_ is negligible.

**Table 1:** Parameters and fitted *p* values for various samples, including single junctions and junction arrays. The superconducting gap Δ of our samples are all about 250 μV (2.9 K).

Sample	*E*_J0_ (K)	*E*_CP_ (K)	*ħω*_p_ (K)	*T* (K)	*p*
JJ1	1.32	0.516	1.17	0.02	2.1~2.4
JJ2	0.82	0.516	0.92	0.02	1.95~2.34
JJ3	4.69	0.028	0.51	0.02	1.96~2.5
JJA (3JJs)	1.49	0.028	0.29	0.02	2.3~2.6
JJA (7JJs)	1.62	0.028	0.30	0.02	1.9~2.6
JJA (13JJs)	5.5	0.028	0.56	0.02	1.95~2.4

**Figure 4:**
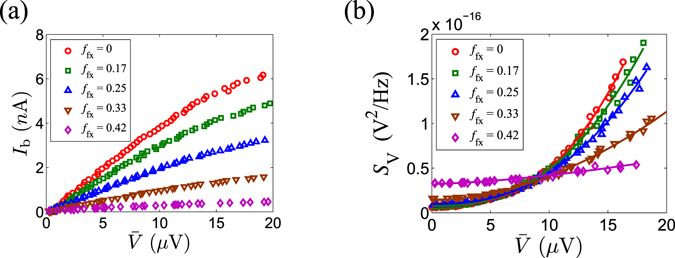
Measurement results (**a**) Measured mean voltage $\bar{V}$ as a function of the bias current *I*_b_ and (**b**) Zero-frequency voltage noise spectrum *S*_V_(0) as a function of mean voltage $\bar{V}$ for sample JJ1. Both plots show curves for flux filling factors *f*_fx_ of 0, 0.17, 0.25, 0.33 and 0.42. Various open symbols are the measured data and the solid curves in (**b**) are power low fitting to the data.

Figure 4(a) and (b) show the DC and noise measurement results, respectively. The quadratic feature of *S*_V_(0) is fitted by a power dependence on the mean voltage $\bar{V}$, *i*.*e*. ${S}_{{\rm{V}}}(0)=a{\bar{V}}^{p}+b$, and the power *p* is summarized in Table 1. It is worth noting that for various single junctions covering a wide range of *E*_J_ and *E*_CP_ parameters, *p* values are all around 2, as expected from Eq. (8). Interestingly, we note that this is also true for Josephson junction arrays.

To understand the quadratic feature of *S*_V_(0) for junction arrays, we propose a conjecture inspired by the phase slip scenario in Josephson junction arrays^19, 20^. Considering an *N*_J_ junction array, the ground state is that each junction equally shares the overall phase difference ${\varphi }_{{\rm{tot}}}$ defined by the mean voltage across the array, that is ${\varphi }_{{\rm{tot}}}/{N}_{{\rm{J}}}$ for each junction. Now, let $|m\rangle$ represent the array state in which phase-particle tunneling takes place in *m* out of *N*_J_ junctions. In these *m* junctions, the phase difference will be $({\varphi }_{{\rm{tot}}}/{N}_{{\rm{J}}})+2\pi$, while it is $({\varphi }_{{\rm{tot}}}/{N}_{{\rm{J}}})-2m\pi /({N}_{{\rm{J}}}-m)$ for the rest. The effective tunneling Hamiltonian describing a transition from $|m\rangle$ to $|m\pm 1\rangle$ is $-{{\rm{\Delta }}}_{{\rm{eff}}}\sum _{m}(|m\rangle \langle m+1|+{\rm{H}}{\rm{.c}}.)$ ^19, 20^, where the effective tunneling strength ${{\rm{\Delta }}}_{{\rm{eff}}}={N}_{{\rm{J}}}{{\rm{\Delta }}}_{0}$. We note that the Hamiltonian for an array and for a single JJ described in Eq. (1) shares the same mathematical structure, and therefore the quadratic feature in the array case is expected based on the proposed analysis.

## Discussions and Conclusions

Last but not least, we would like to make further discussion on the quadratic feature of the voltage shot noise, since it is seemingly contradictive to the generally admitted viewpoint that the quadratic dependence is a feature representing the transition from shot noise to thermal noise in electronic tunnel devices. For a voltage biased tunnel device, the quadratic dependence of the current noise is also due to the imbalance of the bidirectional charge tunneling, namely, $\mathop{{\rm{\Gamma }}}\limits^{\leftharpoonup }={\mathop{{\rm{\Gamma }}}\limits^{\rightharpoonup }e}^{-\beta e{V}_{{\rm{b}}}}$, where $\mathop{{\rm{\Gamma }}}\limits^{\rightharpoonup }$ and $\mathop{{\rm{\Gamma }}}\limits^{\leftharpoonup }$ are the forward and backward tunneling rates, respectively. Although this is similar to the imbalance of the phase particle tunneling ${{\rm{\Gamma }}}_{n+1\to n}={{\rm{\Gamma }}}_{n\to n+1}{{\rm{e}}}^{-\beta {I}_{{\rm{b}}}{{\rm{\Phi }}}_{0}}$, their physical origins are different. In the cases of charge tunneling, the imbalance is partially contributed by energy fluctuation of lead electrons. The influence of this energy fluctuation on the tunneling imbalance is easily understood through the charge tunneling rate $\frac{1}{{e}^{2}{R}_{{\rm{T}}}}{\int }_{-\infty }^{\infty }{\rm{d}}E{\rm{d}}E^{\prime} f(E)[1-f(E^{\prime} +e{V}_{{\rm{b}}})]P(E-E^{\prime} )$ in the absence of energy exchange with the environment (equivalently, $P(E)=\delta (E)$), where *R*_T_ is the tunneling resistance. In this situation, the forward tunneling rate is thus simplified to $\mathop{{\rm{\Gamma }}}\limits^{\rightharpoonup }({V}_{{\rm{b}}})=\frac{1}{{e}^{2}{R}_{{\rm{T}}}}\frac{e{V}_{{\rm{b}}}}{1-{{\rm{e}}}^{-\beta e{V}_{{\rm{b}}}}}$ with the backward tunneling rate being ${\mathop{{\rm{\Gamma }}}\limits^{\rightharpoonup }e}^{-\beta e{V}_{{\rm{b}}}}$ according to $\mathop{{\rm{\Gamma }}}\limits^{\leftharpoonup }({V}_{{\rm{b}}})=\mathop{{\rm{\Gamma }}}\limits^{\rightharpoonup }(-{V}_{{\rm{b}}})$. As discussed in Section 2, by virtue of large separation of intra-well energy levels, the imbalance of the phase particle tunneling is solely due to the detailed balance symmetry of *P*(*E*). This provides the possibility to minimize the system thermal fluctuation under the influence of the environment, resulting in environment-induced shot noise. Indeed, the validity of quadratic feature is verified numerically by plugging in an elevated electron temperature of 100 mK, which is still much smaller than *ħω*_p_, to simulate usual experimental conditions. In summary, we have studied the environment-induced voltage shot noise in Josephson junctions induced by phase particle tunneling. We classified such noise as a new class of shot noise that involves energy exchange with the electromagnetic environment under the condition of *ħω*_p_ $\gg$ *k*_B_ *T*, where the tunneling stochasticity due to the thermal fluctuation of phase particle energy is negligible. We have investigated the theoretical details of the physical picture of such shot noise using the Caldeira-Leggett model for describing the particle-environment interaction and also the *P*(*E*) theory for calculating the tunneling rate as well as the noise spectrum. Based on the analysis, we show that the quadratic dependence of the voltage noise spectrum on the mean voltage is originated from the bias dependent imbalance of the bidirectional tunneling process and is a new feature of shot noise of this kind. This feature is experimentally observed both on single Josephson junctions and junction arrays, verifying the proposed idea of the environment-induced voltage shot noise.

## Electronic supplementary material

Supplementary Information
