# Supplementary material for: Quadratic Characteristics of Environment Induced Voltage Shot Noise in Josephson Junctions
Source: Sci Rep. 2017 Jun 15;7:3567. doi: 10.1038/s41598-017-03790-x (PMC5472641; doi:10.1038/s41598-017-03790-x)
Supplement: Supplementary file 1 — Supplementary Information [file 41598_2017_3790_MOESM1_ESM.pdf]

Supplementary Information for "Quadratic Characteristics of Environment Induced Voltage Shot Noise in Josephson Junctions"

Jyh-Yang Wang, Tao-Hsiang Chung, Teik-Hui Lee, and Chii-Dong Chen

Institute of Physics, Academia Sinica, Taipei 115, Taiwan

In this document, we show the calibration measurement of the voltage thermal noise of a resistor. The measurement setup is verified by the Fano factors extracted from the measured current shot noise. These measurements are described below:

A. Temperature dependence of the voltage noise of a current-biased resistor

To calibrate our voltage noise measurement setup, we measured the voltage thermal noise of a resistor R at varying temperatures and analyzed the data based on the theoretical value of $4k_BTR$ [1-3]. Fig. S1 shows the measurement result (dotted line) and the fitting curve (solid line). The fitting curve is $S_{V,\mathrm{fitting}} = 4ak_BTR + S_{\mathrm{offset}}$ with a correction factor a=0.98 fairly close to unity, and the offset is $S_{\mathrm{offset}} = 4.05\times10^{-18}\,\mathrm{V}^2\cdot\mathrm{Hz}^{-1}$.

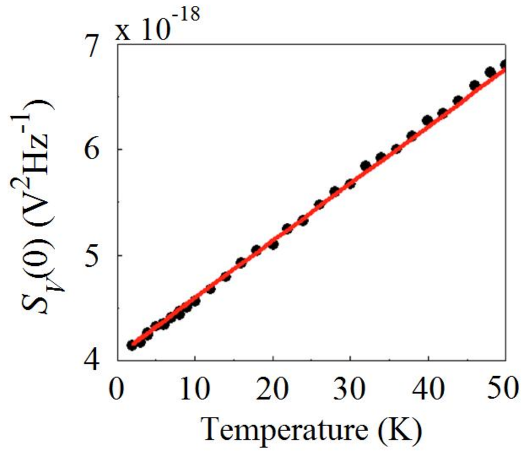

Fig. S1. The voltage thermal noise of a 1kΩ resistor as a function of temperature.

B. Fano factor of the current shot noise

To check to the measurement circuit, we measured the zero-frequency current shot noise spectrum $S_I(0)$ of several voltage-biased aluminum Josephson junction arrays at low temperatures. A magnetic field of 2T was applied to quench the superconductivity of Al, making the devices normal-state tunnel junction arrays. The Fano factor F is analyzed based on $S_I(0) = F\cdot 2eI$, where I is the mean current. The theoretical value

of F of an N-junction array is $1/N$ [4]. Fig. S2(a) shows $S_I(0)$ of a single junction as a function of the mean current I, where the Fano factor equals to unity, verifying the validity of the measurement circuit. Similarly, a Fano factor of 1/3 is obtained for an array of 3 junctions, as shown in Fig S2(b). Table S1 summarizes the Fano factors for arrays of different junction numbers and shows the reliability of the measurement system.

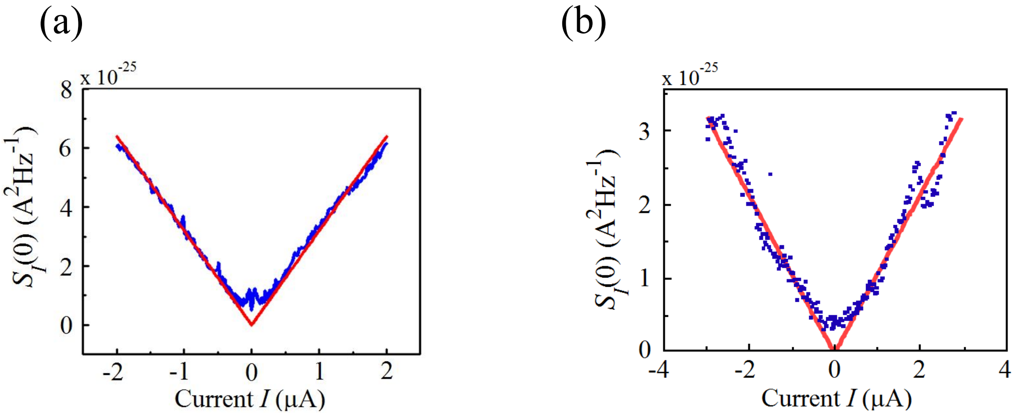

FIG. S2. Zero-frequency current shot noise spectrum $S_I(0)$ as a function of the mean current I of (a) a single junction and (b) a 3-junction array.

Table S1. Fano factors of current shot noise versus array junction numbers.

Junction number of the array	Fano factor F
1	1
3	1/3
7	1/7
13	1/13

References

1. Nyquist, H. Thermal agitation of electric charge in conductors. *Phys. Rev.* **32**, 110 (1928).
2. White, D. R. *et al*. The status of Johnson noise thermometry. *Metrologia* **33**, 325 (1996).
3. Blanter, Y. M. & Büttiker, M. Shot noise in mesoscopic conductors. *Phys. Rep.* **336**, 1 (2000).
4. Korotkov, A. N. & Likharev, K. K. Shot noise suppression at one-dimensional hopping. *Phys. Rev. B* **61**, 15975 (2000).
